# Interplay of serum taurine, S-adenosylmethionine, and cysteine levels in cancer risk: a prospective study

**DOI:** 10.3389/fphar.2024.1507125

**Published:** 2024-12-18

**Authors:** Chenan Liu, Tong Liu, Yaping Wei, Jinyu Shi, Li Deng, Mengmeng Song, Hanping Shi

**Affiliations:** ^1^ Department of Gastrointestinal Surgery, Beijing Shijitan Hospital, Capital Medical University, Beijing, China; ^2^ Department of Clinical Nutrition, Beijing Shijitan Hospital, Capital Medical University, Beijing, China; ^3^ National Clinical Research Center for Geriatric Diseases, Xuanwu Hospital, Capital Medical University, Beijing, China; ^4^ Key Laboratory of Cancer FSMP for State Market Regulation, Beijing, China; ^5^ Laboratory for Clinical Medicine, Capital Medical University, Beijing, China; ^6^ College of Public Health, Shanghai University of Medicine and Health Sciences, Shanghai, China; ^7^ Cardiovascular Research Institute, University of California, San Francisco, CA, United States

**Keywords:** taurine, S-adenosylmethionine, cysteine, cancer, cohort

## Abstract

**Background:**

Amino acids are known to play critical roles in cancer metabolism and progression. Among them, taurine, S-adenosylmethionine (SAM), and cysteine have garnered particular attention due to their interconnected metabolic pathways. This study sought to explore the associations between serum levels of these amino acids and cancer risk within Chinese adults.

**Methods:**

A nested case-control study was conducted within the China H-Type Hypertension Registry Study cohort, comprising 1,391 cancer cases and 1,391 matched controls. Serum concentrations of taurine, SAM, and cysteine were quantified, and their associations with cancer risk were evaluated using conditional logistic regression and Bayesian Kernel Machine Regression (BKMR) models.

**Results:**

A total of 1,391 pairs of participants were included in this study. Their average age was 69.3 years ± 7.77 years, and 56% were male. Higher serum taurine levels were associated with a reduced risk of overall cancer. In contrast, elevated serum SAM levels were linked to an increased risk of digestive cancers. The BKMR model identified complex interactions among these amino acids and showed a significant overall negative association between the combined effect of taurine, SAM, and cysteine and cancer risk.

**Conclusion:**

Serum taurine levels may offer protective benefits against cancer, particularly for digestive cancers, while its metabolites do not have such significant benefits. The intricate interactions among taurine, SAM, and cysteine underscore the need for a comprehensive approach to understanding their roles in the metabolic processes that drive tumorigenesis.

**Clinical Trial Registration:**

https://www.chictr.org.cn/showproj.html?proj=28262, identifier ChiCTR1800017274.

## Introduction

Cancer is a leading cause of morbidity and mortality worldwide, characterized by the uncontrolled proliferation and spread of abnormal cells. Extensive research on cancer incidence and its associated mortality reveals significant public health implications (Siegel et al., 2023). As the global population continues to grow and age, it is anticipated that cancer incidence will increase, further highlighting the urgent need for effective prevention, early detection, and treatment strategies (Mistry et al., 2011). A critical aspect of cancer biology is the metabolic reprogramming that enables cancer cells to sustain their rapid growth and survival. Among the various metabolic pathways involved, non-essential amino acid metabolism is particularly crucial for tumor progression. For example, glutamine, serine, and arginine, though non-essential under normal conditions, are vital for supporting the high proliferation rates of cancer cells (Zhang et al., 2017; Liu T. et al., 2022; Liu et al., 2023). Dysregulations in glucose, fatty acid, and amino acid metabolism pathways are hallmarks of cancer, underscoring the complex relationship between metabolic shifts and tumorigenesis. Gaining a deeper understanding of these metabolic alterations can identify potential therapeutic targets and strategies for combating the disease.

Taurine, a sulfur-containing amino acid, is unique in that it exists in free form in many tissues, particularly in the nervous system, retina, and muscle, rather than being incorporated into proteins. It serves diverse physiological functions, such as acting as a neurotransmitter, regulating calcium homeostasis, and maintaining cell membrane stability (Ping et al., 2023). From a health perspective, taurine is well-known for its antioxidant properties, protecting cells from damage by neutralizing free radicals. It also plays a key role in bile acid conjugation in the liver, thereby aiding in fat digestion and absorption. Beyond these functions, taurine has been linked to cardiovascular health, with evidence suggesting its potential in lowering blood pressure and reducing inflammation (Ridlon et al., 2016). Recent studies have begun to explore taurine’s role in cancer biology. For instance, taurine has been shown to enhance the antitumor efficacy of certain therapies by promoting CD8^+^ T cell function (Ping et al., 2023). However, other studies suggest that taurine metabolism may be involved in cancer progression, indicating that taurine might play a dual role in cancer, depending on the context (Cai et al., 2017). These findings highlight the need for further research to delineate taurine’s specific role and therapeutic potential in cancer.

Taurine, S-adenosylmethionine (SAM), and cysteine are intricately connected through amino acid metabolism. SAM, a universal methyl donor, is involved in numerous biochemical reactions, including the transsulfuration pathway, which leads to cysteine synthesis. Cysteine, in turn, can be converted into several important compounds, including taurine (Brosnan and Brosnan, 2006). This metabolic interrelationship suggests that these amino acids collectively influence various physiological processes. Therefore, analyzing the impact of taurine on cancer risk without accounting for SAM and cysteine levels could result in incomplete or misleading conclusions. Given their complex interactions, a comprehensive analytical approach is essential. In our study, we utilized the Bayesian Kernel Machine Regression (BKMR) model, a nonparametric regression method based on Bayesian statistics, to prospectively evaluate the combined effects of serum taurine and its related amino acids on cancer risk.

## Materials and methods

### Study populations

The participants in this nested case-control research were derived from the China H-Type Hypertension Registry Study (CHHRS; URL: https://www.chictr.org.cn/showproj.html?proj=28262; unique identifier: ChiCTR1800017274). The CHHRS was a real-world observational registry study primarily conducted in Rongcheng city, Shandong Province, China. Running from July 2018 to July 2021, it involved 87,492 participants. Eligible individuals were adults aged 18 and above who were diagnosed with hypertension (defined as SBP ≥ 140 mmHg and/or DBP ≥ 90 mmHg) and had no history of cancer at the time of screening. This study encompassed two main phases: recruitment and a three-year observational follow-up. Recruitment was conducted based on the following inclusion and exclusion criteria. The inclusion criteria were: individuals aged over 18 years, local permanent residents who could cooperate with the required examinations and follow-up, and those who agreed to the study design and signed the informed consent form. The exclusion criteria included: individuals with psychological or nervous system impairments that prevented them from demonstrating informed consent, those unable to comply with follow-up according to the study protocol or planning to relocate in the near future, pregnant individuals, and those in poor health conditions that rendered them unable to cooperate with the investigation and subsequent studies. Follow-ups were organized every 3 months to gather measurement data. These sessions documented parameters such as blood pressure, medication use, and outcomes like cardiovascular disease, cancer, and all-cause mortality.

### Outcome ascertainment

Between 2018 and 2021, cancer cases were identified based on specific clinical criteria. These criteria included surgical records, imaging outcomes, serum tumor markers, and confirmed histopathology data from hospitals treating cancer patients. In the absence of pathological data, two oncologists reviewed potential cases. Both oncologists were required to reach a consensus on the cancer diagnosis, using the International Classification of Diseases, 10th Revision (ICD-10) for coding.

### Nested case-control study design

During the CHHRS follow-up in Rongcheng District, 1,419 new cancer cases were identified among 87,492 participants. An equal number of controls (1,419) were selected from participants who remained cancer-free and alive during the follow-up period (2018–2021). These controls were matched with the cases at a 1:1 ratio based on age, gender, and residency. After excluding individuals without complete data on taurine, SAM, and cysteine, as well as unmatched participants, the final analysis included 1,391 incident cancer cases and their corresponding controls, as detailed in Supplementary Figure 1. The Institute of Biomedicine, Anhui Medical University in Hefei, China, approved both the CHHRS and the present study. All participants provided written informed consent prior to their participation.

### Exposure and covariates

Data on participants’ socioeconomic status, lifestyle habits, medical history, and family medical background were collected through a standardized survey. Height, weight, waist, and hip circumference were measured by trained medical personnel. Venous blood samples were obtained after an overnight fast and collected in EDTA-containing vacuum tubes. Serum concentrations of taurine, SAM, and cysteine were measured using liquid chromatography with tandem quadrupole mass spectrometry (LC-MS/MS) at the Beijing DIAN Medical Laboratory. The specific procedure involved the following steps: first, the samples were prepared by appropriate extraction and purification methods to isolate the target metabolites. Then, the LC-MS/MS system was calibrated with standard solutions of known concentrations of taurine, SAM, and cysteine to ensure accurate quantification. The chromatographic separation was achieved using a specific column and mobile phase conditions optimized for the separation of these metabolites. Mass spectrometry detection was performed in the tandem quadrupole mode, with specific mass-to-charge ratios set for each metabolite to identify and quantify them precisely. Biochemical indicators, including alanine aminotransferase (ALT), triglycerides (TG), total cholesterol (TC), albumin (ALB), high-density lipoprotein cholesterol (HDL-C), fasting blood glucose (FBG), uric acid (UA), and creatinine, were assessed at the Shenzhen Tailored Medical Laboratory using automated clinical analyzers (Beckman Coulter).

### Statistical analysis

Normally distributed variables were presented as mean ± standard deviation (SD), skewed variables as median (interquartile range), and categorical variables as counts and percentages (n, %). Comparisons between cases and controls were conducted using paired Student’s t-tests, nonparametric Kruskal-Wallis tests, or chi-square tests, depending on the distribution of the variables. The dose-response relationships between serum levels of taurine, SAM, and cysteine and the risk of overall, digestive, and non-digestive cancers were evaluated using restricted cubic spline regression (RCS). Conditional logistic regression models were employed to estimate odds ratios (ORs) for cancer incidence associated with continuous and quartile-based serum concentrations of taurine, SAM, and cysteine. The boundaries of the quartiles were population-specific. For example, in the cases of digestive and non-digestive system cancers, we calculated the population-specific quartiles. The models were adjusted for potential confounders, including body mass index (BMI), smoking status, alcohol consumption, antihypertensive drug use, systolic blood pressure SBP, FBG, TC, TG, UA, creatinine, ALT, ALB, and family history of cancer.

To minimize potential reverse causation bias arising from the relatively short follow-up period, the population was categorized based on the median follow-up duration. Further stratified analyses were conducted to evaluate potential interaction effects between serum taurine, SAM, and cysteine levels and cancer risk, with participants categorized into subgroups according to age (median), sex, BMI, and smoking and drinking status. In addition, we also verified the efficacy of different variables in predicting cancer risks through machine learning algorithms and ranked their importance. The models involved include Decision Tree, Lasso Regression, Random Forest, and eXtreme Gradient Boosting (XGboost). Although this is a nested case-control study, we noticed that there were still some variables with differences between the two groups. Therefore, we also conducted propensity score matching (PSM) to verify the relationship between taurine, SAM, and cysteine and cancer risk.

Given the interrelationships among serum taurine, SAM, and cysteine, it is critical to assess whether taurine’s effect on cancer risk remains consistent when considering these metabolites as a combined mixture. To this end, Bayesian Kernel Machine Regression (BKMR) was employed to evaluate the associations between serum taurine, SAM, and cysteine concentrations and cancer risk using the BKMR package in R (Wei et al., 2023). Four parallel Markov Chain Monte Carlo (MCMC) chains were initiated with unique random seeds, each containing 10,000 iterations, with the “family” argument set to “binomial” due to the binary nature of the outcome. Convergence of the BKMR model was confirmed by examining trace, autocorrelation, density, and Gelman-Rubin diagnostic plots. BKMR, which integrates Bayesian statistics with kernel regression, offers the advantage of addressing non-linearities and interactions among predictors, making it superior to traditional logistic regression that assumes linearity and requires explicit interaction terms. Statistical significance was set at a p-value of less than 0.05 for all tests. All analyses were conducted using SAS software (version 9.4) and R (version 4.2.0, https://www.r-project.org).

## Results

### Baseline characteristics

In our research, we examined 1,391 cancer instances, of which 543 were related to the digestive system and 848 were not. The baseline characteristics of the cancer subjects and their corresponding controls are detailed in Supplementary Tables 1, 2. Compared with controls, individuals with cancer displayed reduced ALB and taurine levels, exhibited a higher tendency to smoke, and had an increased incidence of prior coronary heart disease (CHD) and stroke events. It is noteworthy that a higher proportion of cancer patients utilized antihypertensive medications. However, there were no significant disparities in characteristics like age, gender, BMI, blood pressure, ALT, TG, TC, UA, HDL-C, FBG, creatinine, marital status (being married), educational attainment (at least high school), alcohol consumption habits, history of CKD and dyslipidemia, and familial cancer history between the two groups (all p-values > 0.05). The relationships among serum taurine, SAM, and cysteine are illustrated in Supplementary Figure 2. There exists a negative correlation between serum taurine and SAM, and a positive correlation between serum taurine and cysteine. Additionally, serum cysteine is positively correlated with SAM. The correlation coefficients are −0.26 between serum taurine and SAM, 0.15 between serum taurine and cysteine, and 0.24 between serum cysteine and SAM.

### Association of serum taurine, SAM, and cysteine with the risk of incident cancer

**FIGURE 1:**
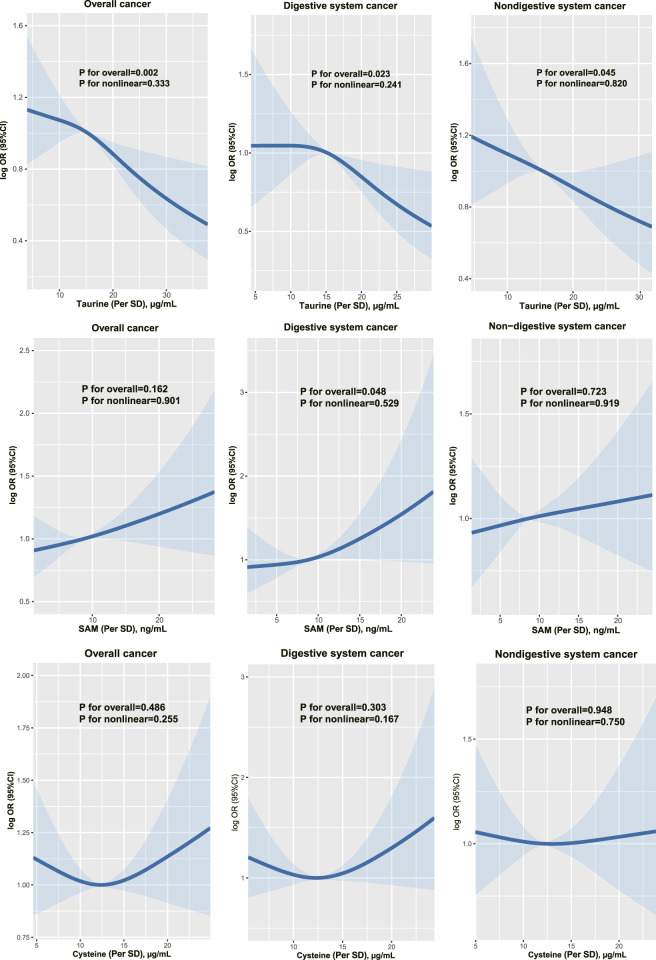
The dose-response associations of taurine, SAM and cysteine with overall, digestive and non-digestive system cancer risk.

Figure 1 shows the dose-response associations of serum taurine (per SD), SAM (per SD), and cysteine (per SD) with the incidence of overall, digestive system, and non-digestive system cancers. Serum taurine is negatively associated with the risk of overall, digestive system, and non-digestive system cancers. Serum SAM is positively associated with the risk of digestive system cancers. Serum cysteine is not significantly associated with the risk of cancer incidence. The associations of serum taurine, SAM, and cysteine levels with cancer risk are presented in Tables 1, 2. After adjusting for other covariates, a continuous increase in serum taurine levels (per SD) was associated with a 16% reduction in the overall risk of cancer (OR = 0.84, 95% CI = 0.75–0.91), a 17% reduction in the risk of digestive system cancers (OR = 0.83, 95% CI = 0.70–0.96), and a 15% reduction in the risk of non-digestive system cancers (OR = 0.85, 95% CI = 0.75–0.96). However, when taurine levels were categorized into quartiles, the highest quartile (Q4) compared to the lowest quartile (Q1) showed a decreased risk of overall cancer.

**TABLE 1:** The association of taurine, SAM, and cysteine with overall cancer risk.

	Cases/controls (ratio 1:1)	Crude model		Adjusted model	
		OR (95%CI)	*p*-value	OR (95%CI)	*p*-value
Taurine (per SD)	1,391/1,391	0.86 (0.78, 0.93)	<0.001	0.84 (0.75, 0.91)	<0.001
Quartiles of taurine (μg/mL)^a^					
Q1	368/327	Ref.		Ref.	
Q2	350/344	0.89 (0.71, 1.11)	0.287	0.91 (0.71, 1.17)	0.497
Q3	341/357	0.83 (0.66, 1.03)	0.090	0.80 (0.61, 1)	0.048
Q4	332/363	0.78 (0.62, 0.98)	0.034	0.72 (0.53, 0.92)	0.007
*P* for trend		0.028		0.002	
SAM (per SD)	1,391/1,391	1.09 (1.00, 1.19)	0.051	1.07 (0.96, 1.19)	0.208
Quartiles of SAM (ng/mL)^b^					
Q1	351/383	Ref.		Ref.	
Q2	350/333	1.15 (0.94, 1.42)	0.411	1.20 (0.95, 1.51)	0.125
Q3	330/352	1.05 (0.84, 1.30)	0.123	1.14 (0.89, 1.46)	0.300
Q4	360/323	1.26 (1.00, 1.59)	0.050	1.27 (0.97, 1.67)	0.085
*P* for trend		0.107		0.124	
Cysteine (per SD)	1,391/1,391				
Quartiles of cysteine (μg/mL)^c^		1.03 (0.93, 1.13)	0.580	1.01 (0.90, 1.13)	0.747
Q1	355/340	Ref.		Ref.	
Q2	335/362	0.88 (0.70, 1.10)	0.265	0.85 (0.67, 1.09)	0.220
Q3	349/346	0.97 (0.75, 1.26)	0.831	0.97 (0.71, 1.31)	0.936
Q4	352/343	0.99 (0.76, 1.31)	0.963	0.95 (0.70, 1.30)	0.786
*P* for trend		0.647		0.392	

Note: Models were adjusted for taurine, S-adenosylmethionine, body mass index, smoking status, alcohol drinking, systolic blood pressure, triglycerides, cholesterol, uric acid, fasting blood glucose, high-density lipoprotein cholesterol, creatinine, albumin, alanine aminotransferase, cysteine, sleep quality, antihypertensive drug usage, and family history of cancer.

^a^ Quartile for taurine: 0 < Q1<11.72 μg/mL ≤ Q2<15.34 μg/mL ≤ Q3<19.14 μg/mL ≤ Q4.

^b^ Quartile for SAM: 0 < Q1<5.72 μg/mL ≤ Q2<8.69 μg/mL ≤ Q3<11.89 μg/mL ≤ Q4.

^c^ Quartile for cysteine: 0 < Q1<8.40 μg/mL ≤ Q2<12.26 μg/mL ≤ Q3<15.84 μg/mL ≤ Q4.

**TABLE 2:** The association of taurine, SAM, and cysteine with the digestive and non-digestive system cancer risk.

	Digestive system cancer		Non-digestive system cancer	
	Cases/controls	OR (95%CI)	Cases/controls	OR (95%CI)
Taurine (per SD)	543/543	0.83 (0.70, 0.96)	848/848	0.85 (0.75, 0.96)
Quartiles of taurine^a^				
Q1	143/128	Ref.	224/199	Ref.
Q2	140/132	0.97 (0.64, 1.40)	210/215	0.85 (0.62, 1.17)
Q3	127/144	0.79 (0.50, 1.17)	216/210	0.84 (0.61, 1.17)
Q4	133/139	0.80 (0.52, 1.26)	198/224	0.72 (0.52, 1.00)
*P* for trend	0.061		0.072	
SAM (per SD)	543/543	1.21 (1.01, 1.44)	848/848	1.02 (0.90, 1.16)
Quartiles of SAM^b^				
Q1	133/150	Ref.	216/232	Ref.
Q2	137/131	1.02 (0.70, 1.50)	214/204	1.26 (0.93, 1.70)
Q3	130/138	1.20 (0.80, 1.80)	204/211	1.15 (0.83, 1.58)
Q4	143/124	1.33 (0.85, 2.08)	214/201	1.19 (0.84, 1.70)
*P* for trend	0.561		0.271	
Cysteine (per SD)^c^	543/543	1.02 (0.84, 1.22)	848/848	1.01 (0.88, 1.17)
Quartiles of cysteine^c^				
Q1	136/134	Ref.	219/207	Ref.
Q2	136/137	0.94 (0.60, 1.43)	201/222	0.80 (0.58, 1.11)
Q3	132/140	0.97 (0.59, 1.60)	214/208	0.98 (0.66, 1.43)
Q4	139/132	0.94 (0.55, 1.61)	214/211	1.02 (0.69, 1.49)
*P* for trend	0.794		0.531	

Note: All the OR, values were the adjusted OR, values. Models were adjusted for taurine, S-adenosylmethionine, body mass index, smoking status, alcohol drinking, systolic blood pressure, triglycerides, cholesterol, uric acid, fasting blood glucose, high-density lipoprotein cholesterol, creatinine, albumin, alanine aminotransferase, cysteine, sleep quality, antihypertensive drug usage, and family history of cancer.

^a^ The cutoffs of taurine in the digestive system were 11.74, 15.19, and 18.90.

The cutoffs of taurine in the non digestive system were 11.74, 15.19, and 18.90.

^b^ The cutoffs of SAM, in the digestive system were 5.68, 8.82, and 11.94.

The cutoffs of SAM, in the non digestive system were 5.74, 8.62, and 11.86.

^c^ The cutoffs of cysteine in the digestive system were 8.51, 12.47, and 15.92.

The cutoffs of cysteine in the nondigestive system were 8.33, 12.15, and 15.83.

To avoid reverse causation and eliminate the impact of relatively short follow-up time in this study, we further grouped the subjects based on the median follow-up time (Supplementary Table 3). It is worth noting that the impacts of serum taurine and SAM on cancer incidence became evident only in the subgroup observed beyond the median follow-up time. Specifically, we observed that serum taurine (per SD increase) and its quartiles (Q4 vs. Q1) were associated with a decreased overall cancer risk, with odds ratios (95% confidence intervals) of 0.81 (0.71, 0.92) and 0.67 (0.47, 0.97), respectively. In contrast, serum SAM (Q3 vs. Q1) was associated with an increased overall cancer risk, with an OR (95% CI) of 1.51 (1.07, 2.13). In addition, different machine learning models also confirm the relationship between taurine and cancer risk. In the Decision Tree, Random Forest, and XGBoost, taurine ranks first in terms of importance (Supplementary Figure 3). Meanwhile, we also carried out a PSM analysis. The matched cohort contained data of 1,021 pairs of participants, and the baseline characteristics are shown in Supplementary Table 4. Conditional logistic regression indicated that for each standard deviation increase in taurine, the cancer risk decreased by 17% (95% CI: 10%–28%) (Supplementary Table 5).

**FIGURE 2:**
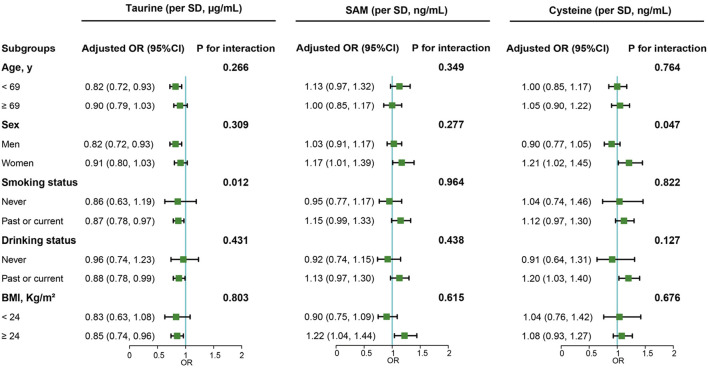
Subgroup analyses of the associations of taurine, SAM and cysteine with overall cancer risk. Note: Models were adjusted for taurine, S-adenosylmethionine, body mass index, smoking status, alcohol drinking, systolic blood pressure, triglycerides, cholesterol, uric acid, fasting blood glucose, high-density lipoprotein cholesterol, creatinine, albumin, alanine aminotransferase, cysteine, sleep quality, antihypertensive drug usage, and family history of cancer.

In our subgroup analysis (Figure 2), we noted a consistent protective effect of serum taurine against cancer risk in several demographic groups: males, individuals aged under 69 (median), current/former smokers, current/former drinkers, and those classified as obese. Notably, the protective influence of serum taurine against cancer was significantly modified by smoking habits (interaction P value < 0.05). For female and obese populations, there were discernible positive associations between serum SAM levels and heightened cancer risk. Furthermore, elevated levels of serum cysteine corresponded to a higher cancer risk among female subjects and among those who are current or former drinkers. The impact of serum cysteine on cancer risk was found to be significantly influenced by sex (interaction P value < 0.05).

### Analyzing micronutrient mixtures using BKMR

**FIGURE 3:**
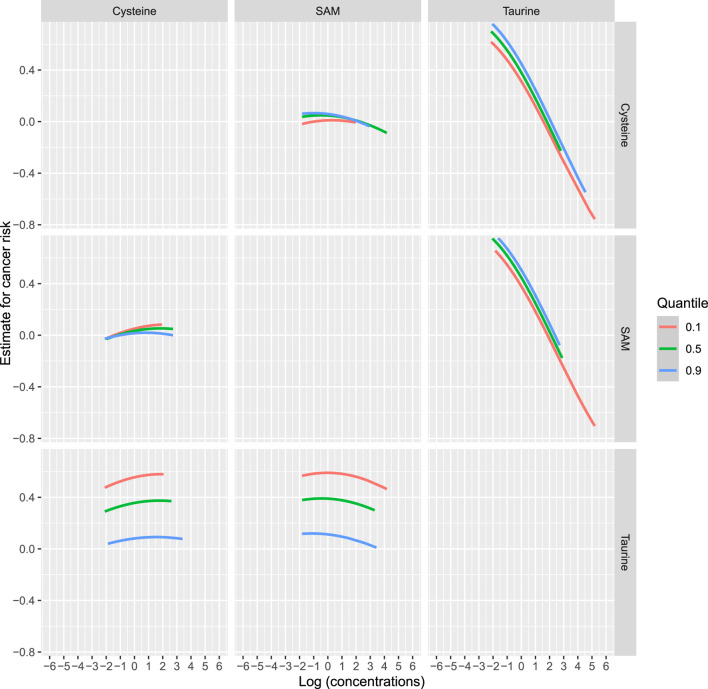
Bivariate exposure dose-response curves for taurine, SAM, and cysteine. Note: Dose-response curves for one variable at the 10th, 50th, and 90th percentile levels of the other variable (the remaining two variables were all fixed at the median) versus outcome. The *x*-axis represents the logarithmic values of the concentrations of taurine, SAM, and cysteine, and the *y*-axis represents the estimate for cancer risk.

**FIGURE 4:**
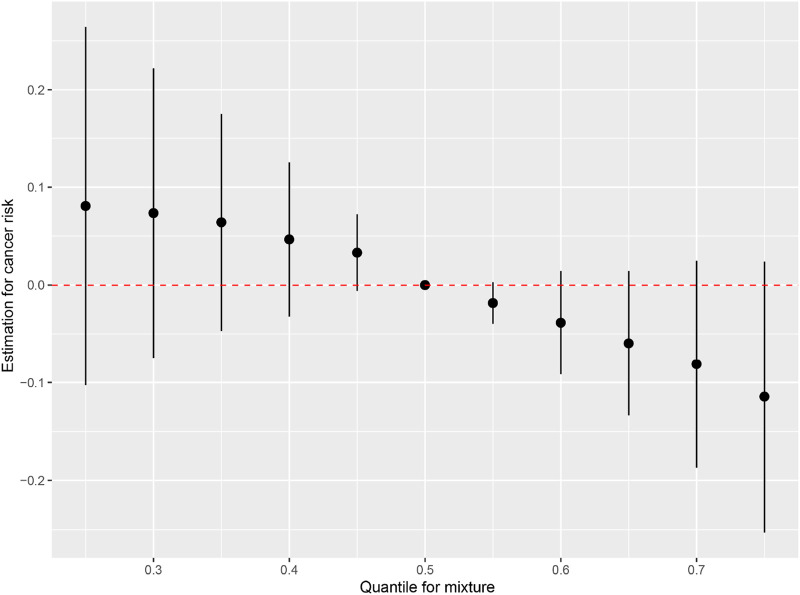
The association between the overall effect of the mixture and cancer risk. Note: The change in the estimated outcome when the three compounds were fixed at different percentiles simultaneously compared to when they were fixed at the median shows that the overall effect is negatively correlated with the outcome.

To comprehensively account for the intercorrelations among taurine, SAM, and cysteine, as well as their associations with incident cancer, we employed BKMR methodology. Supplementary Figure 4 illustrates the exposure-response relationship between a singular variable and the outcome, while holding the levels of the other two variables constant at their respective median values. The primary objective of this analysis is to investigate the nonlinear correlation between the exposure variable and the outcome. After controlling for the levels of SAM and cysteine, there is a negative correlation between serum taurine and the risk of overall cancer incidence. Figure 3 illustrates the bivariate dose-response functions of taurine, SAM, and cysteine concerning the risk of incident cancer. Within each panel, the principal variable of interest is designated at the uppermost section of the figure. Meanwhile, the second predictor, denoted on the side of the figure, is held constant at the 10th, 50th, and 90th percentiles, while the remaining predictor is fixed at its median value. These curves depict the overall cancer risk across continuous values of the primary exposure at the specified percentiles of the secondary exposure. This analysis unveils that the relationships between SAM and cysteine with overall cancer risk may be contingent upon the levels of taurine. Conversely, the inverse associations observed between taurine and cancer risk remain unaffected by the levels of SAM and cysteine. Figure 4 demonstrates the association between the overall effect of the mixture (taurine, SAM, and cysteine) and overall cancer risk. When the three micronutrients are held constant at different percentiles, they can be compared to when they are set at their respective medians. This comparison reveals a significant negative association between the combined effect of taurine, SAM, and cysteine and the overall cancer risk.

## Discussion

In this large-scale population-based case-control study nested within CHHRS, we identified a robust correlation between serum levels of taurine, SAM, and cysteine. Notably, taurine demonstrated a protective effect, decreasing the overall risk of cancer incidence. This effect was observed for both digestive and non-digestive system cancers. In contrast, elevated SAM levels were associated with a heightened risk for gastrointestinal cancers. Subgroup analyses revealed that increased serum cysteine concentrations were associated with a higher incidence of cancers, particularly in women and the obese population. Within the context of the BKMR model, the oncogenic effects of serum cysteine and SAM were found to be influenced by taurine concentrations. Intriguingly, increased combined levels of these three micronutrients were concomitantly linked to a reduced overall cancer risk.

To the best of our understanding, this represents the first population-based prospective study examining the impact of serum taurine, SAM, and cysteine on the risk of incident cancers. Nonetheless, several studies partially corroborate our results:

Our findings align with the observation that serum taurine is inversely associated with the incidence of overall, digestive and non-digestive cancers. This association is evident in males under 69 years, smokers, drinkers, and the obese participants. Several epidemiological studies have highlighted a potential link between taurine consumption and a reduced risk of certain cancers. For instance, research has shown that populations with high seafood consumption, a primary source of taurine, have a lower prevalence of certain conditions, including cancer (Murakami, 2015). Moreover, a study focusing on urinary biomarkers identified elevated taurine levels in the urine of bladder cancer patients, suggesting its potential as a biomarker for non-muscle invasive bladder cancer (Srivastava et al., 2010). Furthermore, taurine has been proposed as a novel tumor marker for enhanced detection of breast cancer, emphasizing its significance in cancer diagnostics (El et al., 2011). The antioxidant properties of taurine, which play a pivotal role in neutralizing oxidative stress—a key factor in tumorigenesis—further underscore its potential therapeutic value (Jong et al., 2021).

Our findings reveal that taurine demonstrates a protective effect against cancer risk, with this effect varying between different smoking populations. Specifically, while no significant protective effect of taurine was observed in non-smokers, a pronounced protective effect was evident in smokers. This interaction can be attributed to the oxidative stress induced by smoking. Smoking introduces a myriad of reactive oxygen species (ROS) and other harmful agents, leading to oxidative stress. Taurine, known for its antioxidative properties, may mitigate some oxidative damage induced by smoking. Research has shown that taurine can modify monocyte and endothelial dysfunction in smokers, potentially mitigating the vascular damages caused by smoking-related oxidative stress (Shorey-Kendrick et al., 2024). Thus, the pronounced protective effect of taurine in smokers might be due to its enhanced antioxidative action countering the elevated oxidative stress in this group.

We found that elevated serum SAM was linked to a higher incidence of digestive cancers. Additionally, it showed a positive association with overall cancer incidence in females and individuals with a BMI over 24 kg/m^ 2. Dysregulation of SAM metabolism can lead to aberrant methylation patterns, which have been implicated in the initiation and progression of various cancers. While the direct epidemiological evidence linking SAM to cancer risk remains limited, several studies have highlighted the importance of methylation processes in cancer development and progression (Garcia-Martinez et al., 2021). For instance, hypermethylation of tumor suppressor genes or hypomethylation of oncogenes can drive carcinogenesis, and SAM, as a primary methyl donor, plays a central role in these processes (Roadmap et al., 2015). Alterations in SAM levels can also affect substrates’ availability for polyamine synthesis, a pathway known to influence cancer cell growth and proliferation (Casero et al., 2018).

Although we observed no direct link between serum cysteine and overall cancer incidence, there was a significant risk in subgroups of women and drinkers. Elevated cysteine levels have been associated with an increased risk of specific cancers. An epidemiological study has observed a positive correlation between cysteine levels and breast cancer risk (Lin et al., 2010). Another study highlighted that the oxidized form of the OGG1-S326C polymorphic variant, which has a cysteine substitution at position 326, is associated with an increased risk of cancer, as observed in several molecular epidemiological studies (Soliman et al., 2020).

Our research has revealed a gender-specific variation in the pro-cancer effect of serum cysteine. Specifically, while no significant effect was observed in males, a pronounced risk of cancer was evident in females. This gender-specific interaction may be rooted in the differential metabolic pathways and hormonal environments between males and females. For instance, estrogen, a predominant female hormone, has been shown to influence various metabolic processes, potentially modulating the effects of cysteine and other metabolites (Yu et al., 2022). Additionally, a study has indicated that oxidative stress markers, including those related to cysteine metabolism, can vary with smoking habits and are influenced by sex (Oda et al., 2022). Thus, the heightened risk observed in females may stem from unique metabolic and hormonal interactions that are less pronounced or absent in males.

While traditional regression provides an initial understanding of individual associations, it may not adequately capture the complex interactions and combined effects of these nutrients on cancer risk. Therefore, we employed BKMR, a more advanced analytical method, to assess the collective impact on tumorigenesis and potential interactions among these three micronutrients. Specifically, the relationship between SAM, cysteine, and cancer risk is influenced by taurine levels. Yet, taurine’s protective effect against cancer remains steady regardless of SAM and cysteine concentrations. SAM is a primary methyl donor in cellular reactions. Aberrant methylation patterns, especially in DNA, can lead to the silencing of tumor suppressor genes or activation of oncogenes, thereby promoting tumorigenesis (Mattei et al., 2022). The presence of taurine might influence both SAM’s availability and its methylation potential, each of which could modulate its impact on cancer risk (Pepe et al., 2007; Li and Ye, 2020). Cysteine is a precursor to the antioxidant glutathione. An imbalance in the redox state can lead to oxidative stress, a known contributor to cancer initiation and progression (Chen et al., 2020). Taurine, with its antioxidative properties, might synergize or antagonize with cysteine, influencing the overall redox balance and, consequently, cancer risk (Gulcin, 2020). Our findings indicated a negative association between serum taurine levels and cancer risk, while serum SAM and cysteine levels showed a positive association with cancer susceptibility. Given the intertwined relationships among these biomolecules, their combined influence on cancer risk is of paramount interest. To elucidate this collective impact, we utilized the BKMR model. This model uniquely facilitates the assessment of the overall effect of a mixture on an outcome, capturing the nuanced interactions among multiple components. Our results suggested a potential trend that the combined effect of these three factors might be associated with a certain degree of protection against cancer as their concentrations increase. However, it should be noted that while the BKMR model was applied in our study to explore the relationships among these factors, the evidence for a clear and strong synergistic amplification of the combined protective effect is not yet conclusive. The BKMR model does provide a useful framework to analyze the complex relationships and conditional effects among multiple exposures, which contributes to a more comprehensive understanding of the possible interactions involved in cancer etiology, although further research is needed to more precisely define and validate these relationships (Devick et al., 2022).

The intricate mechanisms behind taurine’s tumor-suppressive effects, and the tumor-promoting roles of SAM and cysteine, are subjects of ongoing research. Taurine, which is known for its antioxidative properties, may potentially play a role in counteracting oxidative stress, which is an aspect that has been associated with cancer initiation (Jakaria et al., 2019), but also modulate cellular inflammation, further hindering tumor progression (Li et al., 2022). SAM, central to methylation processes, can influence gene expression. Aberrant methylation, especially of tumor suppressor genes, can drive carcinogenesis (Chi et al., 2023), and SAM might also affect epigenetic modifications, further amplifying cancer susceptibility (Herceg, 2016). Cysteine, when imbalanced, can disrupt redox homeostasis, leading to the production of ROS and interacting with cellular pathways to promote tumorigenesis (Liu S. et al., 2022). Additionally, cysteine’s role in modulating certain proteases has implications for both cancer progression and therapeutic outcomes (Jakoš et al., 2019). These insights emphasize the multifaceted roles of amino acids in oncology and the need for continued research in this domain.

The primary advantage of this study lies in its novel insight into the potential tumor-inhibiting properties of taurine and the tumor-enhancing roles of SAM and cysteine within Chinese adults. Furthermore, the prospective design minimizes recall bias and is optimally tailored for analyzing time-to-event data. However, the present study has certain limitations worth mentioning. First, we assessed serum taurine, SAM, and cysteine levels only at the outset. Dynamic evaluations might have provided a clearer perspective on the evolving associations between these amino acids and cancer susceptibility. Second, although we employed the BKMR model to handle the complex interactions between taurine and its metabolites with the aim of enhancing the robustness of the results, the limited number of cancer cases and the relatively short observation period have impeded an in-depth analysis of cancer subtypes. This emphasizes the need for a larger sample size to conduct further validation and draw more reliable conclusions. Third, our study primarily addressed the prevalence and outcomes of H-type hypertension in China, centering on hypertensive individuals. The generalizability of our findings to normotensive populations is yet to be ascertained, although we did account for blood pressure variations in our multivariate analysis, minimizing its potential impact. Fourth, while we identified associations of taurine, SAM, and cysteine with cancer risk, the causative implications of elevated amino acid levels, or their significance in the synthesis of cancer-associated compounds, require deeper exploration. Lastly, given that our results are based on a nested case-control design, they underscore the need for further research through comprehensive cohort studies and randomized trials.

## Conclusion

Our findings establish the prospective role of taurine in cancer prevention and highlight the combined effects of taurine, SAM, and cysteine levels on cancer incidence in Chinese adults. Should our results be corroborated in future investigations, they might offer a novel, effective, and safe avenue for cancer prevention.

## Data Availability

The raw data supporting the conclusions of this article will be made available by the authors, without undue reservation.
