# Supplementary material for: Interplay of serum taurine, S-adenosylmethionine, and cysteine levels in cancer risk: a prospective study
Source: Front Pharmacol. 2024 Dec 18;15:1507125. doi: 10.3389/fphar.2024.1507125 (PMC11688464; doi:10.3389/fphar.2024.1507125)
Supplement: Supplementary file 1 [file Table1.docx]

**Supplementary Tables and Figures**

**Supplementary Table 1. Baseline characteristics of the cases and matched controls.**

| Variables | Controls  (n=1,391) | Cancer Cases  (n=1,391) | *p*-value |
| --- | --- | --- | --- |
| Age, y | 69.30±7.77 | 69.30±7.77 | 0.999 |
| Male, n (%) | 779 (56.00) | 779 (56.00) | 1.000 |
| BMI, kg/m^2^ | 25.73±3.60 | 25.73±3.83 | 0.950 |
| ALB, g/L | 45.44±2.45 | 44.75±2.97 | <0.001 |
| TC, mmol/L | 6.51±1.23 | 6.44±1.30 | 0.045 |
| HDL-C, mmol/L | 1.23±0.24 | 1.22±0.27 | 0.243 |
| FBG, mmol/L | 6.25±1.71 | 6.29±1.83 | 0.613 |
| Creatinine, μmol/L | 51.0 (10.0, 64.0) | 52.0 (10.0, 64.0) | 0.752 |
| ALT, U/L | 10.0 (7.0, 13.0) | 10.0 (7.0, 14.0) | 0.140 |
| UA, μmol/L | 320.0 (269.0, 371.0) | 314.0 (264.0, 374.0) | 0.389 |
| TG, mmol/L | 1.21 (0.86, 1.77) | 1.19(0.84, 1.80) | 0.641 |
| Marital status, n (%) | 1146 (82.39) | 1180 (84.83) | 0.354 |
| Educational background, n (%) | 109 (7.84) | 103 (7.40) | 0.668 |
| Current smoker, n (%) | 334 (24.01) | 401 (28.83) | 0.011 |
| Current drinker, n (%) | 388 (27.89) | 370 (26.60) | 0.359 |
| History of CKD, n (%) | 14 (1.01) | 25 (1.80) | 0.076 |
| History of CHD, n (%) | 0 (0) | 165 (11.86) | <0.001 |
| History of Stroke, n (%) | 0 (0) | 64 (4.60) | <0.001 |
| History of dyslipidemia, n (%) | 129 (9.27) | 131 (9.42) | 0.896 |
| Family history of cancer, n (%) | 47 (3.38) | 50 (3.59) | 0.918 |
| Antihypertensive drug usage, n (%) | 495 (35.59) | 557 (40.04) | 0.015 |

Note: BMI: body mass index; SBP: systolic blood pressure; DBP: diastolic blood pressure; ALT: alanine aminotransferase; ALB: albumin; TG: triglycerides; TC: total cholesterol; UA: uric acid; HDL-C: High-density lipoprotein cholesterol; FBG: Fasting blood glucose; CKD: chronic kidney disease; CHD: coronary heart disease.

**Supplementary Table 2. Serum taurine, SAM and cysteine levels in this cohort.**

| Variables | Controls  (n=1,391) | Cancer Cases  (n=1,391) | *p*-value |
| --- | --- | --- | --- |
| Taurine, μg/mL | 15.63 (11.95,19.38) | 15.11 (11.53,18.99) | 0.035 |
| SAM, ng/mL | 8.54 (5.58, 11.66) | 8.81 (5.87, 12.06) | 0.077 |
| Cysteine, μg/mL | 12.16 (8.44, 15.82) | 12.32 (8.35, 15.93) | 0.856 |

**Note: SAM: S-adenosyl methionine**

**Supplementary Table 3. The** **association of taurine with overall cancer risk stratified by the median follow-up.**

|  | Before the median follow-up | | |  | After the median follow-up | |
| --- | --- | --- | --- | --- | --- | --- |
|  | Cases/controls | | OR (95%CI) |  | Cases/controls | OR (95%CI) |
| Taurine (per SD) | 699/699 | | 0.93 (0.81, 1.06) |  | 692/692 | 0.81 (0.71, 0.92) |
| Quartiles of taurine ^a^ |  | |  |  |  |  |
| Q1 | 179/172 | | Ref. |  | 190/156 | Ref. |
| Q2 | 183/167 | | 1.11 (0.78, 1.58) |  | 168/178 | 0.75 (0.53, 1.06) |
| Q3 | 166/182 | | 0.91 (0.63, 1.29) |  | 174/173 | 0.77 (0.54, 1.10) |
| Q4 | 171/178 | | 0.95 (0.66, 1.37) |  | 160/185 | 0.67 (0.47, 0.97) |
| *P* for trend | 0.687 | | |  | 0.048 | |
| SAM (per SD) | 699/699 | | 1.07 (0.91, 1.26) |  | 692/692 | 1.05 (0.92, 1.21) |
| Quartiles of SAM ^b^ |  | |  |  |  |  |
| Q1 | 180/181 | | Ref. |  | 169/200 | Ref. |
| Q2 | 181/166 | | 1.08 (0.77, 1.50) |  | 170/169 | 1.26 (0.91, 1.76) |
| Q3 | 156/189 | | 0.83 (0.58, 1.20) |  | 179/161 | 1.51 (1.07, 2.13) |
| Q4 | 182/163 | | 1.00 (0.67, 1.49) |  | 174/162 | 1.36 (0.93, 2.01) |
| *P* for trend | 0.561 | | |  | 0.068 | |
| Cysteine (per SD) ^c^ | 699/699 | 1.11 (0.95, 1.30) | |  | 692/692 | 0.93 (0.79, 1.09) |
| Quartiles of cysteine ^c^ |  |  | |  |  |  |
| Q1 | 169/182 | Ref. | |  | 183/161 | Ref. |
| Q2 | 174/175 | 1.11 (0.76, 1.63) | |  | 168/180 | 0.81 (0.56, 1.17) |
| Q3 | 168/181 | 1.17 (0.76, 1.79) | |  | 179/167 | 0.81 (0.56, 1.17) |
| Q4 | 188/161 | 1.48 (0.94, 2.33) | |  | 162/184 | 0.68 (0.43, 1.07) |
| *P* for trend | 0.313 | | |  | 0.101 | |

Note: Models were adjusted for taurine, S-adenosylmethionine, body mass index, smoking status, alcohol drinking, systolic blood pressure, triglycerides, cholesterol, uric acid, fasting blood glucose, high-density lipoprotein cholesterol, creatinine, albumin, alanine aminotransferase, cysteine, sleep quality, antihypertensive drug usage, and family history of cancer.

a: The cutoffs of taurine in the group before median follow-up were 11.84, 15.41, and 19.26.

The cutoffs of taurine in the group after median follow-up were 11.61, 15.14, and 19.00.

b: The cutoffs of SAM in the group before median follow-up were 5.73, 8.69, and 11.84.

The cutoffs of SAM in the group after median follow-up were 5.69, 8.67, and 11.94.

c: The cutoffs of cysteine in the group before median follow-up were 8.52, 12.57, and 16.00.

The cutoffs of cysteine in the group after median follow-up were 8.31, 11.82, and 15.71.

Results presented with bold values were statistically significant.

**Supplementary Table 4 Baseline characteristics of the cases and matched controls after PSM.**

| Variables | Controls  (n=1021) | Cancer Cases  (n=1021) | *p*-value |
| --- | --- | --- | --- |
| Age, y | 62.19±5.17 | 62.19±5.17 | 0.999 |
| Male, n (%) | 535(52.4) | 535(52.4) | 1.000 |
| BMI, kg/m^2^ | 25.01±2.39 | 25.01±2.39 | 0.961 |
| ALB, g/L | 45.31±2.22 | 45.29±2.31 | 0.841 |
| TC, mmol/L | 6.39±1.29 | 6.41±1.37 | 0.733 |
| HDL-C, mmol/L | 1.22±0.21 | 1.22±0.31 | 0.997 |
| FBG, mmol/L | 6.26±1.64 | 6.27±1.74 | 0.989 |
| Creatinine, μmol/L | 51.0 (10.0, 64.0) | 52.0 (10.0, 64.0) | 0.782 |
| ALT, U/L | 10.0 (7.0, 13.0) | 10.0 (7.0, 14.0) | 0.223 |
| UA, μmol/L | 319.0 (269.0, 371.0) | 314.0 (264.0, 374.0) | 0.430 |
| TG, mmol/L | 1.31 (0.93, 1.86) | 1.33(0.93, 1.96) | 0.919 |
| Marital status, n (%) | 842(82.47) | 855(83.74) | 0.443 |
| Educational background, n (%) | 75(7.35) | 75(7.35) | 1.000 |
| Current smoker, n (%) | 255 (24.98) | 264 (25.86) | 0.209 |
| Current drinker, n (%) | 271 (26.54) | 270 (26.44) | 0.960 |
| History of CKD, n (%) | 11 (1.08) | 19 (1.86) | 0.141 |
| History of CHD, n (%) | 0 (0) | 120 (11.75) | <0.001 |
| History of Stroke, n (%) | 0 (0) | 58 (5.68) | <0.001 |
| History of dyslipidemia, n (%) | 101 (9.89) | 107 (10.48) | 0.661 |
| Family history of cancer, n (%) | 41 (4.02) | 45 (4.41) | 0.659 |
| Antihypertensive drug usage, n (%) | 413 (40.45) | 437 (42.80) | 0.281 |

Note: BMI: body mass index; SBP: systolic blood pressure; DBP: diastolic blood pressure; ALT: alanine aminotransferase; ALB: albumin; TG: triglycerides; TC: total cholesterol; UA: uric acid; HDL-C: High-density lipoprotein cholesterol; FBG: Fasting blood glucose; CKD: chronic kidney disease; CHD: coronary heart disease.

**Supplementary Table 5. The association of taurine, SAM, and cysteine with overall cancer risk after PSM.**

|  | Adjusted model | |
| --- | --- | --- |
|  | OR (95%CI) | p-value |
| Taurine (per SD) | 0.83(0.72, 0.90) | <0.001 |
| Quartiles of taurine (μg/mL) |  |  |
| Q1 | Ref. |  |
| Q2 | 0.88 (0.70, 1.09) | 0.202 |
| Q3 | 0.79 (0.61, 0.96) | 0.043 |
| Q4 | 0.72 (0.57, 0.86) | 0.001 |
| P for trend | 0.001 | |
| SAM (per SD) | 1.04 (0.94, 1.15) | 0.211 |
| Quartiles of SAM (ng/mL) |  |  |
| Q1 | Ref. |  |
| Q2 | 1.18 (0.92, 1.49) | 0.337 |
| Q3 | 1.12 (0.85, 1.40) | 0.419 |
| Q4 | 1.17 (0.92, 1.53) | 0.168 |
| P for trend | 0.206 | |
| Cysteine |  |  |
| Quartiles of cysteine (μg/mL) | 1.04 (0.92, 1.17) | 0.692 |
| Q1 | Ref. |  |
| Q2 | 0.82 (0.63, 1.06) | 0.107 |
| Q3 | 1.01 (0.78, 1.36) | 0.923 |
| Q4 | 0.95 (0.69, 1.30) | 0.827 |
| P for trend | 0.555 | |

Note: Models were adjusted for taurine, body mass index, smoking status, alcohol drinking, systolic blood pressure, triglycerides, cholesterol, uric acid, fasting blood glucose, high-density lipoprotein cholesterol, creatinine, albumin, alanine aminotransferase, homocysteine, sleep quality, antihypertensive drug usage, and family history of cancer.

**Supplementary Figure 1. Flow chart of study participants in the nested case-control study within the CHHRS.**

**
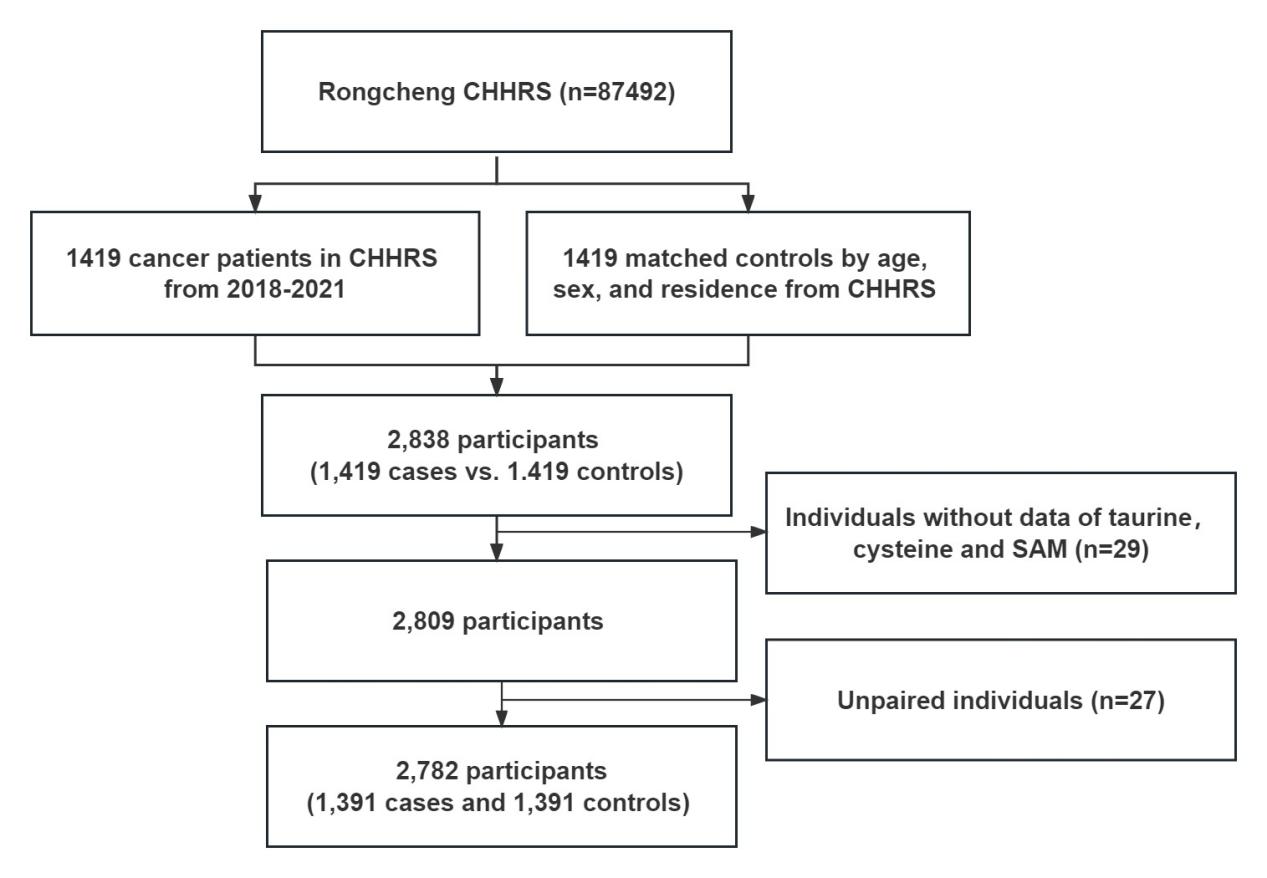
**

**Supplementary Figure 2. The correlations between taurine, SAM and cysteine.**

**
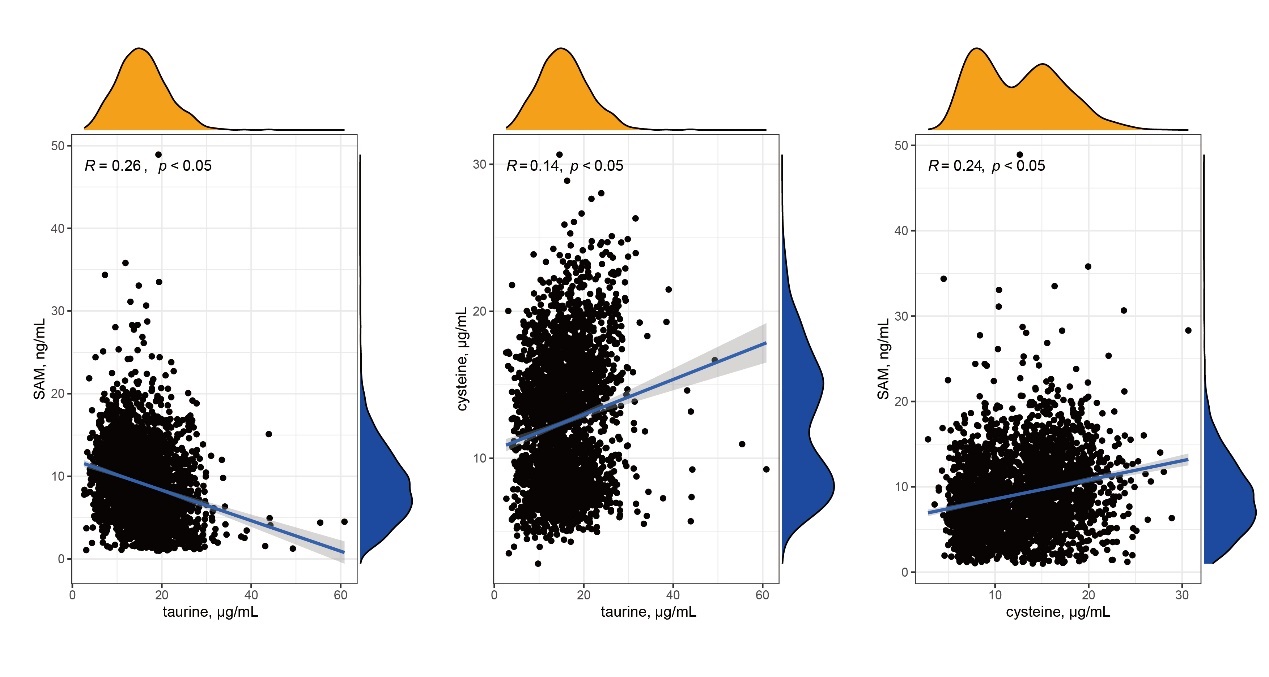
**

**Supplementary Figure 3. Ranking of the Importance of Different Factors (Top 10) under Different Machine Learning Models**


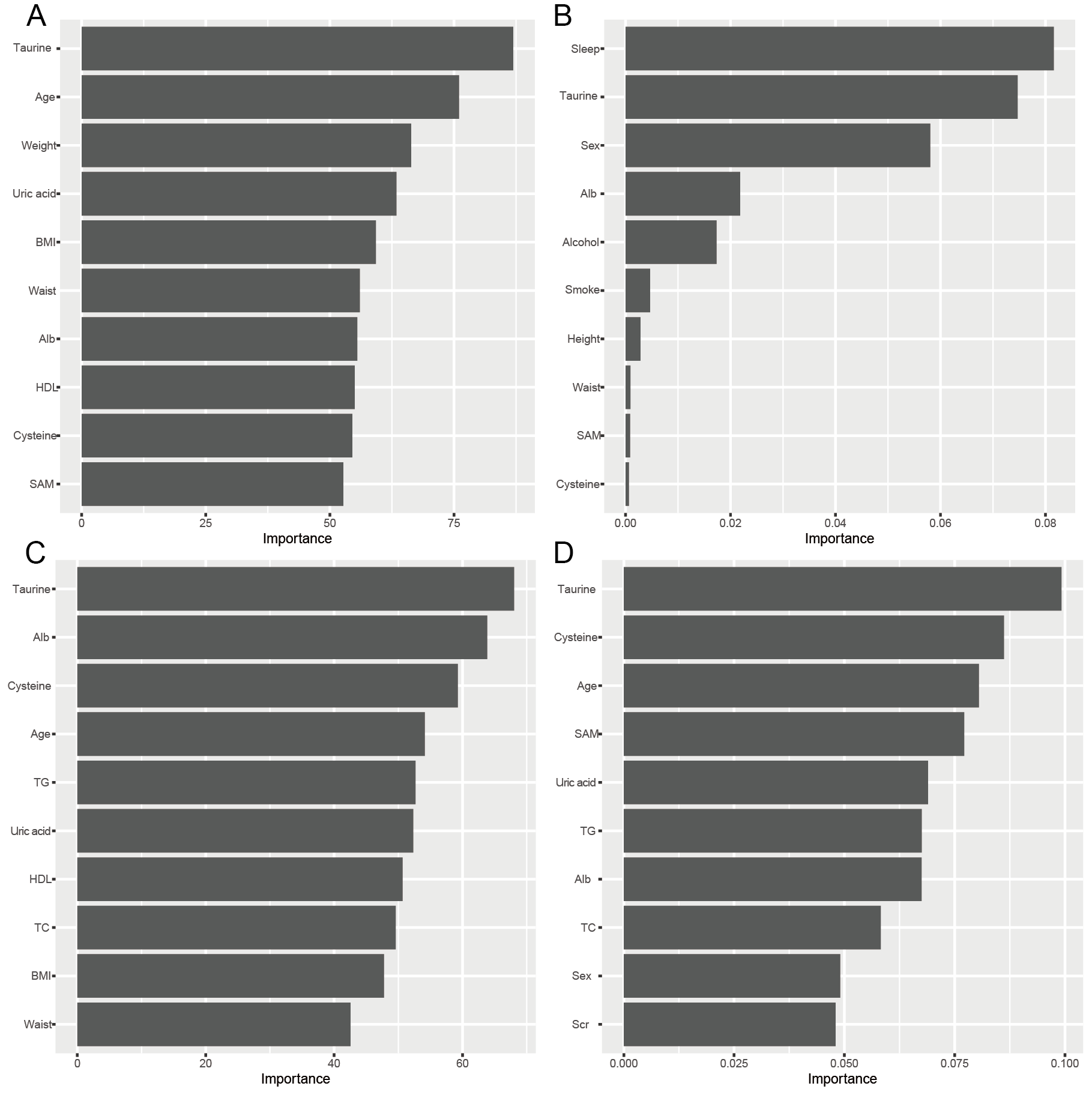


Note: A : Decision tree, B: Lasso regression, C: Random forest, D: XGboost

**Supplementary Figure 4. Univariate cross-section of taurine, SAM, and cysteine**

**
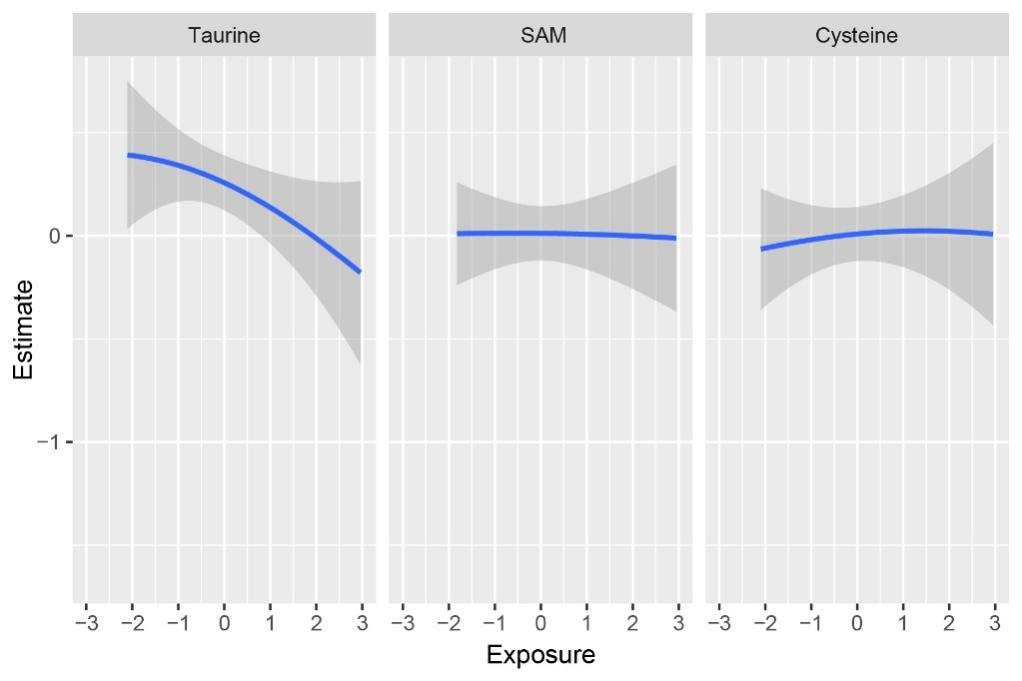
**

Note: Exposure-response cross sections for a single variable versus outcome when fixing the levels of the remaining 2 variables fixed at the median, mainly to see the nonlinear association between exposure and outcome.
